# A Clustering-Based Approach to Functional and Biomechanical Parameters Recorded with a Pair of Smart Eyeglasses in Older Adults in Order to Determine Physical Performance Groups

**DOI:** 10.3390/s24051427

**Published:** 2024-02-22

**Authors:** Justine Hellec, Serge S. Colson, Amyn Jaafar, Olivier Guérin, Frédéric Chorin

**Affiliations:** 1 Université Côte d’Azur, LAMHESS, France; justine.hellec@univ-cotedazur.fr (J.H.); chorin.f@chu-nice.fr (F.C.); 2 Ellcie Healthy, 06600 Antibes, France; 3 Université Côte d’Azur, CHU, France; jaafar.a@chu-nice.fr (A.J.); guerin.o@chu-nice.fr (O.G.); 4 Université Côte d’Azur, CNRS, INSERM, IRCAN, France

**Keywords:** fall, frailty, sit-to-stand, timed up and go, unsupervised analysis, inertial measurement unit

## Abstract

Falls and frailty status are often associated with a decline in physical capacity and multifactorial assessment is highly recommended. Based on the functional and biomechanical parameters measured during clinical tests with an accelerometer integrated into smart eyeglasses, the purpose was to characterize a population of older adults through an unsupervised analysis into different physical performance groups. A total of 84 participants (25 men and 59 women) over the age of sixty-five (age: 74.17 ± 5.80 years; height: 165.70 ± 8.22 cm; body mass: 68.93 ± 13.55 kg) performed a 30 s Sit-to-Stand test, a six-minute walking test (6MWT), and a 3 m Timed Up and Go (TUG) test. The acceleration data measured from the eyeglasses were processed to obtain six parameters: the number of Sit-to-Stands, the maximal vertical acceleration values during Sit-to-Stand movements, step duration and length, and the duration of the TUG test. The total walking distance covered during the 6MWT was also retained. After supervised analyses comparison (i.e., ANOVAs), only one of the parameters (i.e., step length) differed between faller groups and no parameters differed between frail and pre-frail participants. In contrast, unsupervised analysis (i.e., clustering algorithm based on K-means) categorized the population into three distinct physical performance groups (i.e., low, intermediate, and high). All the measured parameters discriminated the low- and high-performance groups. Four of the measured parameters differentiated the three groups. In addition, the low-performance group had a higher proportion of frail participants. These results are promising for monitoring activities in older adults to prevent the decline of physical capacities.

## 1. Introduction

The progressive decline in most physiological systems with aging has a negative impact on the health and well-being of older adults. The musculoskeletal system is a major determinant of reduced functional physical capacities, which could lead to frailty and/or falls. Frailty is defined as “a physiological vulnerability related to the intrinsic aging of the person” [1] and it affects ~18% of older adults in Europe [2]. The World Health Organization [3] defined a fall as “an event which results in a person coming to rest inadvertently on the ground or floor or other lower level”. This phenomenon affects one third of the persons over the age of 65 [4,5] and fifty percent over the age of 85 [6,7,8,9]. The consequences of frailty or falls are damaging for older adults, impairing their quality of life and eventually leading to a loss of autonomy or even dependence [10]. The consequences are also economic, with higher medical costs for frail and falling older adults [11,12,13] making frailty and falls major public health concerns.

Although the two are distinct, there is recent evidence that falls are associated with frailty in older adults [14,15] and both frailty and falls should be assessed in people aged 60 years and over. Frailty status is generally evaluated with the physical frailty phenotype consisting of three questions (i.e., unintentional weight loss, general fatigue, physical activity level) and two measures of functional physical capacities (i.e., grip strength, walking speed) [11]. The incidence of falls can be assessed by a simple question like “have you fallen in the last 12 months?” [16]. To provide a more objective characterization of the person’s risk of falling, this question can be combined with a measure of functional physical capacities through different clinical tests that reproduce daily life movements. For example, among these tests, the 30 s Sit-to-Stand (STS) test measures lower limb muscle strength and endurance [17], the six-minute walk test (6MWT) assesses walking capacity in older adults [18], and the Timed Up and Go (TUG) test was developed to evaluate functional mobility [19]. Although these tests have been used to discriminate fallers from non-fallers [20,21,22,23], it is extremely challenging to determine precisely whether an older adult is at risk of falling using a simple test [24]. Consequently, multifactorial fall risk assessment is highly recommended in the literature [21,22,25].

Due to the complexity and non-linearity that characterizes the clinical status of individuals, an alternative approach was recently used to categorize the frailty or faller status in older adults from multifactorial measures [26,27]. This approach is based on unsupervised clustering analysis that aims at identifying similarities in clinical outcomes within a population. For example, blood analyses (e.g., total cholesterol, fasting glucose, hemoglobin, hematocrit) and both waist and arm circumferences were measured based on their capacity to better categorize frailty in older adults [26]. Similarly, the categorization of the risk of falling was determined from different clinical tests (i.e., TUG, functional reach test, and handgrip strength) and cardiovascular data (e.g., systolic and diastolic blood pressure variability ratio and heart rate variability ratio) [27]. The authors obtained four groups corresponding to physical performance levels that were associated with different levels of fall risk. Considering the non-linearity observed in frailty development [26], as well as faller status [27], this approach seems more relevant to categorizing older adults according to different physical performance levels.

The emergence of wearable technologies [28] now enables the monitoring of daily life movements by inertial measurement units (IMUs) that acquire data from accelerometers and gyroscopes integrated into everyday objects (e.g., watches, smartphones, etc.). IMUs have the advantage of being small, low-cost, portable, and user friendly [29]. Some disadvantages can include a lack of acceptance for fall detection assessment [30,31] and a lack of consensus on the placement of IMUs. Although most of the studies placed the IMUs close to the body’s center of mass or at the wrist [32], some studies have suggested that head-level placement improves movement accuracy for fall detection compared to wrist [33] or hip and trunk [34] placements. In this context, connected smart eyeglasses have been developed and validated to monitor Sit-to-Stand movements and spatiotemporal parameters during walking in healthy young participants [35,36]. However, to date, smart eyeglasses embedding an IMU have not been used to assess the functional physical capacities of older adults. Smart eyeglasses are unobtrusive, can be worn comfortably throughout the day, and provide a non-invasive way to monitor physical capacities. In addition, the acceptability of these eyeglasses for fall detection assessment in older adults seems higher than other devices (i.e., watches and necklaces) [37]. It is therefore of major interest to test the ability of smart eyeglasses to measure the functional and biomechanical parameters of daily life movements performed in clinical settings.

Therefore, the purpose of this study was to assess functional physical capacities (i.e., 30 s STS test, 6MWT, and TUG test) in older adults with the use of smart eyeglasses. The first objective of this study was to assess the dependence of frailty and faller status on selected functional and biomechanical parameters measured with the eyeglasses. The second objective was to characterize the population through a clustering approach based on these parameters. We hypothesized that participants would be categorized in different physical performance group levels independently of their frailty or faller status.

## 2. Materials and Methods

### 2.1. Particpants

This observational study included a convenience sample of 84 volunteer participants (25 men and 59 women; aged 74.17 ± 5.80 years, height 165.70 ± 8.22 cm; body mass 68.93 ± 13.55 kg; body mass index 25.07 ± 4.40 kg/m^2^; mean ± standard deviation). Inclusion criteria: aged 65 years and over, able to walk without any aid (e.g., canes, walkers), and autonomous in daily living activities. Participants were informed of the experimental procedures. Informed consent was obtained from all the participants. This study was approved by the South Mediterranean Protection of Persons Ethics Committee (registration number: 2015-A01188-41) and conducted according to the revised Declaration of Helsinki of 2013.

### 2.2. Experimental Design

Participants were required to attend the laboratory (Plateforme fragilité, Nice University Hospital Center, Nice, France) on a single occasion between October 2020 and October 2021. To evaluate fall history, a clinician asked the participant “Have you fallen in the last 12 months?”. Participants were considered fallers if their answer was “yes” [16]. Frailty status was determined from Fried’s frailty phenotype [11] and participants were classified as robust, pre-frail, or frail. Participants were considered frail when they met at least three of five frailty criteria (unintentional body mass loss, muscle weakness, general fatigue, slowed walking speed, and low physical activity). Three functional physical capacity tests were performed in the same order for all participants: (i) the 30 s Sit-to-Stand (STS) test, (ii) the 6 min walk test (6MWT), and (iii) the 3 m Timed Up and Go (TUG) test. The tests were performed when the participants had fully understood the instructions of each test to ensure each test was carried out correctly (e.g., the experimenter performed a demonstration and the participants could perform practice trials). In order to address potential sources of bias associated with the clinical evaluations, only highly skilled and trained experimenters hired by the University Hospital Center performed the assessment of the participants’ functional physical capacities. The evaluations performed are used in clinical routine in the University Hospital Center and standardized realization instructions were given to the participants. During all tests, participants were equipped with smart eyeglasses without correction (Ellcie-Healthy, Antibes, France). The experimenter had to adjust eyeglasses on the nose and behind the ears of the participant and determine that he/she had clearly understood the instructions before the test. Once equipped with smart eyeglasses, participants were free to decide whether to continue with the tests or not. All older adults performed the 30 s STS, the 6MWT, and the 3 m TUG test (i.e., compliance with evaluations was 100%). In addition, there was no harm associated with these tests to report in this study.

### 2.3. Apparatus, Data Collection

**Figure 1:**
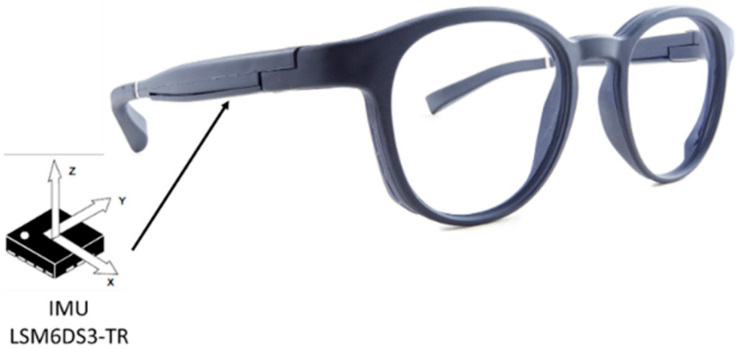
Placement of the Inertial Measurement Unit (IMU) in the temple of the smart eyeglasses.

The smart eyeglasses used in this study were embedded with an IMU (Inertial Measurement Unit; LSM6DS3-TR; 2.5 mm × 3 mm × 0.83 mm), combining an accelerometer and a gyroscope in the right temple (Figure 1). The inertial units are 3-dimensional and are oriented forward along the anterior–posterior *y*-axis towards the left along the medio-lateral *x*-axis and upward along the vertical *z*-axis. The range of measurement was set at ±8 g for the accelerometer and ±250°/s for the gyroscope. The sampling rate of the IMU was 26 Hz. Eyeglasses were connected to a smartphone (Samsung A5) through a Bluetooth connection. Raw data from both the accelerometer and the gyroscope were sent to the mobile application (Research, Ellcie-Healthy, Antibes, France) and were saved on the smartphone. Data were processed offline with MATLAB scripts (MathWorks, Inc., Natick, MA, USA, version R2018a) that were created in order to derive the different parameters of interest for each test. Before the beginning of each test, participants had to maintain a static position for 10 s to clearly detect the onset of motion from the accelerometer signal.

During the 30 s STS test, participants had to perform the maximum possible number of STS movements with their arms crossed on their chest from a starting position sitting on a chair (standard height of 45 cm without armrests). During the test, two parameters were measured with the smart eyeglasses: (i) the total number of complete STS movements [38] and (ii) the average of the maximal vertical acceleration values recorded during the STS movements [35]. The peaks of maximal vertical acceleration provided by the IMU were detected during offline analysis [35] and the number of STS movements realized over the 30 s period was obtained from these peaks.

During the 6MWT, participants had to cover the greatest possible distance in 6 min, without running, in a 10 m shuttle. Besides the total distance obtained from the number of shuttles performed [18], two previously validated spatiotemporal parameters were collected with the smart eyeglasses [36]. During offline analysis, step duration was first calculated from each step, represented by the temporal difference between two consecutive maximal vertical acceleration peaks recorded by the smart eyeglasses’ IMU. Second, the step length was deduced from step duration and the number of steps realized in each 10 m. The average of all step durations and all step lengths computed was used for statistical analyses.

The 3 m TUG test consisted of standing up from the standardized 45 cm chair, walking 3 m, turning round, walking back the 3 m, and sitting down on the chair. This test was carried out in two conditions: (i) at a comfortable walking speed and (ii) at the fastest possible walking speed without running. In both conditions, the ground was flat without obstacles. The total time to complete the test was obtained during offline analysis thanks to the IMU of the smart eyeglasses detecting the moments when the participant stood up and sat down again at the beginning and end of the test (i.e., onset and termination of movement).

### 2.4. Statistical Analysis

Through the different physical tests, besides the distance covered during the 6MWT, six functional and biomechanical parameters were obtained from the smart eyeglasses for our older adults (i.e., number of STS, maximal vertical acceleration during the STS, average step duration during the 6MWT, average step length during the 6MWT, TUG time in comfort condition, TUG time in fast condition). Since this study included a convenience sample of older adults, there were no missing data. A Shapiro–Wilk test was used to assess the normality of the data distribution. Participants were allocated to one of two groups, depending on their fall history (i.e., fallers and non-fallers). The same procedure was carried out for the frailty phenotype, with participants divided into three groups (i.e., robust, pre-frail, and frail). Considering the first objective, aiming at assessing the influence of faller and frailty status on the selected functional and biomechanical parameters measured, we compared these parameters between the faller and non-faller groups and between the three frailty groups. Between-group comparisons were performed with one-way ANOVAs for normally distributed data and with Mann–Whitney U tests (two groups) or Kruskall–Wallis tests (three groups) for non-normally distributed data. The effect size of the ANOVAs was estimated from partial eta square (η^2^p) values and considered small when ~0.01, medium when ~0.06, and large when ≥0.14. A post hoc Tuckey HSD test for unequal sample sizes was used to compare means. Fall history proportion was assessed using k proportions tests to compare frailty phenotypes.

The second objective was to create physical performance groups from the functional and biomechanical parameters measured. An unsupervised analysis was performed to determine the optimal number of clusters and to allocate the participants to the different groups of physical performance. First, a clustering algorithm was created in Python (version 2.7.18) based on K-means (unsupervised analysis) [27]. Second, the optimal number of clusters was identified from the maximum silhouette coefficient [39]. For cluster determination, the initial centroids in each group were selected randomly, and each participant was assigned to the closest centroid by calculating the Euclidean distance. The initial positions were refined during the analysis to create centroids that were the most spaced in terms of distance from each other, and to have the smallest distance between participants in the same group to centroids [27]. Participants were then assigned to different distinct groups of physical performance based on the coefficient score and a performance equation was obtained through linear regression of the functional and biomechanical parameters. Demographic data, and the functional and biomechanical parameters of the performance groups created, were analyzed with one-way ANOVAs for normal data distribution and with Kruskall–Wallis tests for non-normally distributed data. Sex, fall history, and Fried’s phenotype proportions were compared using k proportions tests to compare the different performance groups. Statistica (Statsoft, version 8.0 Tulsa, OK, USA) and XLSTAT (Addinsoft, version 2022.1, Paris, France) software was used for analyses and the significance level was set at *p* < 0.05. Throughout the manuscript, unless specified, data are expressed as mean ± standard deviation in the tables and figures.

## 3. Results

### 3.1. Fall History Groups

The faller group comprised 34 participants (age 74.50 ± 5.94 years; height 166.00 ± 8.26 cm; body mass 70.61 ± 14.20 kg; 24 females and 10 males) and the non-faller group comprised 50 participants (age 73.95 ± 5.76 years; height 165.50 ± 8.27 cm; body mass 67.79 ± 13.11 kg; 34 females and 16 males). Except for the average step length (F(1,82) = 4.24, *p* < 0.05, η^2^p = 0.05), no significant difference between groups was observed for any of the variables (Table 1). A post hoc analysis indicated that the average step length in the faller group tended to be shorter than in the non-faller group (*p* = 0.06).

### 3.2. Frailty Phenotype Groups

Based on Fried’s frailty phenotype, 24 participants were robust (age 72.90 ± 5.87 years; height 164.29 ± 7.96 cm; body mass 65.63 ± 10.68 kg; 20 females and 4 males), 45 were pre-frail (age 73.75 ± 5.67 years; height 166.00 ± 8.64 cm; body mass 71.34 ± 15.15 kg; 29 females and 16 males), and the remaining 15 participants were frail (age 77.48 ± 5.20 years, height 167.07 ± 7.48 cm; body mass 67.00 ± 11.74 kg; 9 females and 6 males). According to the frailty phenotype, the proportion of fallers was 60%, 42.2%, and 25% in the frail, pre-frail, and robust groups, respectively. No significant difference was found in the proportion of fallers between groups (χ^2^ = 4.82, *p* = 0.09).

Significant differences were observed for the number of STS performed (F(2,81) = 8.30, *p* < 0.001, η^2^p = 0.17), the average maximal vertical acceleration during the STS (F(2,81) = 7.30, *p* < 0.01, η^2^p = 0.15), the total distance covered (F(2,81) = 5.75, *p* < 0.01, η^2^p = 0.12), and the average step length (F(2,81) = 3.61, *p* < 0.05, η^2^p = 0.08) during the 6MWT (Table 2). The TUG time in the fast condition also significantly differed (H(2) = 7.45, *p* = 0.024). The robust group performed a higher number of STS (*p* < 0.01), reached a greater average maximal vertical acceleration during the STS (*p* < 0.01), walked a longer distance during the 6MWT (*p* = 0.01) with a wider average step length (*p* < 0.05) than the frail group, and completed the TUG faster in fast condition (*p* = 0.02).

The average step duration (F(2,81) = 2.14, *p* = 0.12, η^2^p = 0.05) and TUG time during the comfort condition (F(2,81) = 2.74, *p* = 0.07, η^2^p = 0.06) did not differ significantly between groups. Although a significant effect was noted for age (F(2,81) = 3.31, *p* < 0.05, η^2^p = 0.08), a post hoc analysis did not reveal significant differences between groups.

### 3.3. Physical Performance Groups

**Figure 2:**
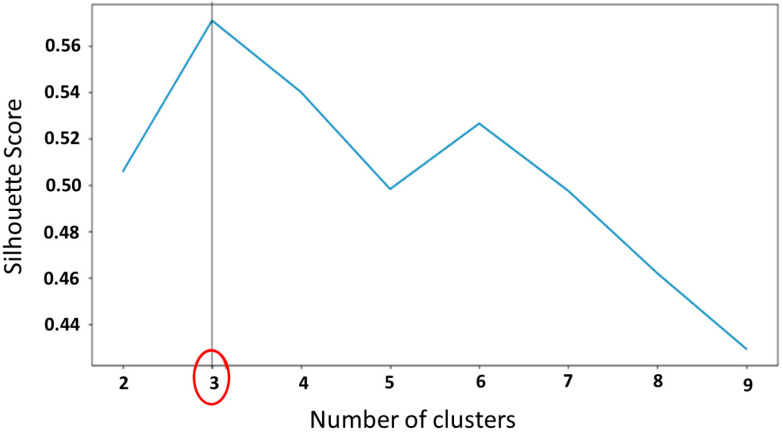
Graph of silhouette method to determine the optimal number of clusters for the functional and biomechanical parameters measured. The red circle indicates the number of clusters chosen.

To classify participants into different groups, the optimal number of clusters was first determined through the maximum silhouette score (Figure 2). The K-means clustering algorithm was used to achieve the best clustering performance through adjusting the centroids. The recommended number of clusters for the population included in this study, and the functional and biomechanical parameters analyzed, was three, which allowed us to create three distinct groups.

**Figure 3:**
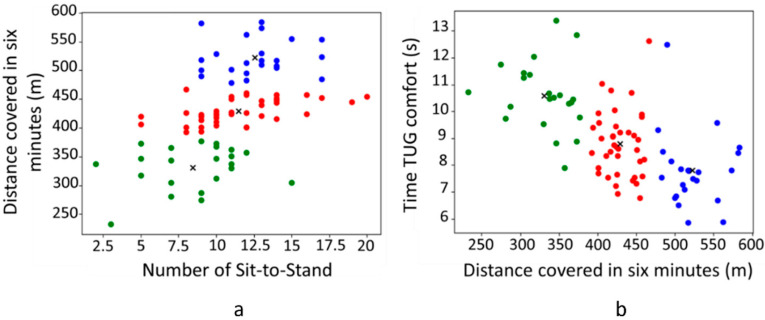
K-means clustering of the three clusters (n = 84). Representation of the physical performance between the distance covered in 6 min and the number of Sit-to-Stand (**a**) and between the TUG time in comfort condition and the distance covered in 6 min (**b**). Green dots represent the low physical performance group (LPP; n = 22); red dots represent the intermediate physical performance group (INT; n = 39); and blue dots represent the high physical performance group (HPP; n = 23). Black crosses represent the centroids of each group.

To illustrate this classification, in Figure 3, two examples of the distribution of participants in the three performance groups is shown, for (i) the distance covered during the 6MWT with the maximum number of STS and (ii) the time to complete the TUG in the comfort condition with the distance covered during the 6MWT. The participants allocated to the green group covered a short distance during the 6MWT, performed a low number of STS, and needed a longer time to complete the TUG in comfort condition. The participants in the blue group performed more STS than the other two groups, covered a greater distance during the 6MWT, and completed the TUG in the comfort condition more rapidly. Finally, the participants in the red group had intermediate values between the two other groups. A level of physical performance was then assigned for the three groups as follows: (i) the green group—“low physical performance-LPP”, (ii) the red group—“intermediate physical performance-IPP”, and (iii) the blue group—“high physical performance-HPP”.

#### 3.3.1. Characteristics of the Three Performance Groups

After the clustering procedure, the three physical performance groups were compared regarding demographic data, fall history, and Fried’s phenotype.

Significant age differences were found between groups (F(2,81) = 15.91, *p* < 0.001, η^2^p = 0.28). HPP participants were younger than the IPP and LPP participants (*p* < 0.001; Table 3). No significant difference was found between the IPP and LPP groups (*p* = 0.27). Height (F(2,81) = 2.03, *p* = 0.14), body mass (F(2,81) = 0.02, *p* = 0.98), and the proportions of males and females (χ^2^ = 1.57, *p* = 0.46) were not different between groups. The percentage of fallers in the last 12 months did not significantly differ between the three groups (χ^2^ = 0.54, *p* = 0.76). Regarding frailty phenotype, while no difference was found between groups in the proportions of robust (χ^2^ = 3.83, *p* = 0.15) and pre-frail (χ^2^ = 3.46, *p* = 0.18) participants, the proportion of frail participants significantly differed (χ^2^ = 10.82, *p* = 0.004). The LPP group had a higher proportion of frail participants compared to both the IPP and HPP groups (Table 3).

#### 3.3.2. Physical Capacities of the Three Performance Groups

**Figure 4:**
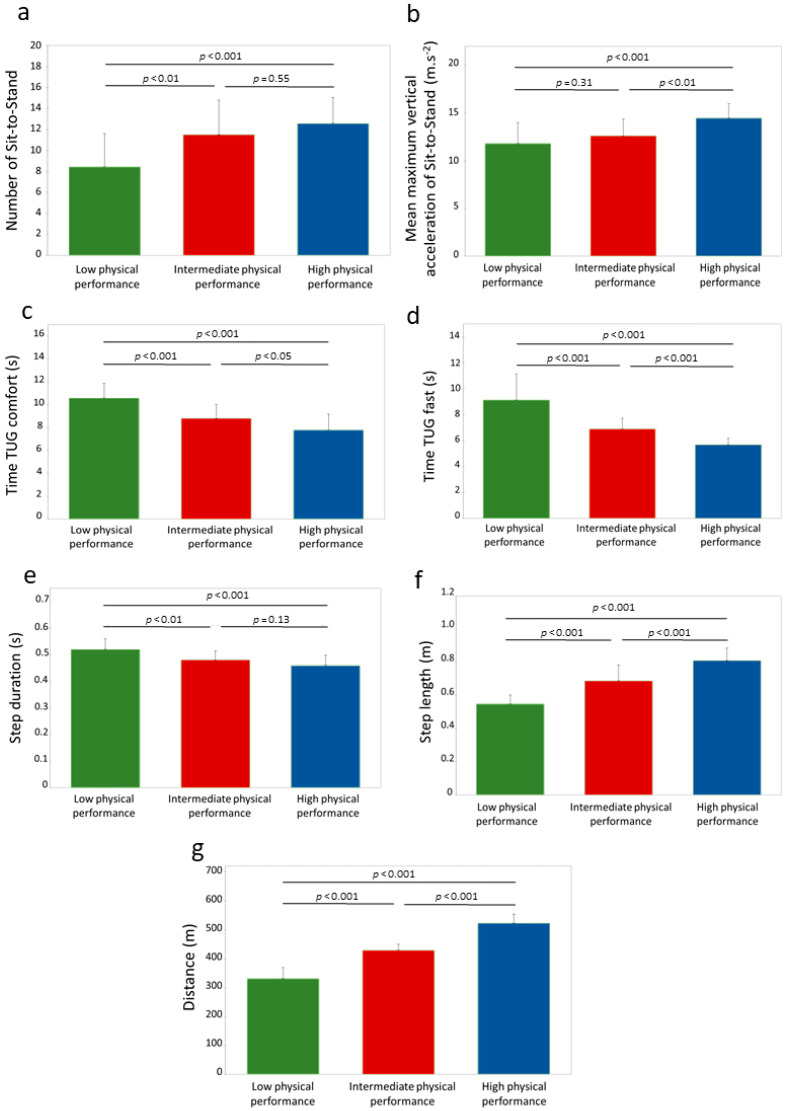
Differences of functional and biomechanical parameters measured between the three performance groups (n = 84). (**a**) Number of Sit-to-Stand; (**b**) mean maximum vertical acceleration of Sit-to-Stand; (**c**) time of TUG comfort; (**d**) time of TUG fast; (**e**) step duration; (**f**) step length and; (**g**) distance. Times of fast TUG were analyzed through Kruskall–Wallis test and ANOVAs were used for all other parameters. Significant differences between performance groups are indicated by horizontal lines and *p* values.

The number of STS (F(2,81) = 11.08, *p* < 0.001, η^2^p = 0.22) and the average maximal vertical acceleration during the STS (F(2,81) = 13.08, *p* < 0.001, η^2^p = 0.24) significantly differed between the physical performance groups. The number of STS was lower in the LPP group compared with the IPP (*p* < 0.01) and the HPP (*p* < 0.001) groups. No significant difference was found between the IPP and HPP groups (*p* = 0.55). The average maximal vertical acceleration during the STS was significantly higher in the HPP group compared to the IPP (*p* < 0.01) and LPP (*p* < 0.001) groups. No significant difference was noted between the LPP and IPP groups (*p* = 0.31) (Figure 4a,b). 

The time to complete the TUG significantly differed between groups in both the comfort (F(2,81) = 27.09, *p* < 0.001, η^2^p = 0.40) and fast conditions (H(2) = 55.64, *p* < 0.001). The LPP group performed the TUG more slowly than the IPP and HPP in both conditions (*p* < 0.001). The HPP completed the test faster than the IPP in comfort (*p* < 0.05) and in fast (*p* < 0.001) conditions (Figure 4c,d). 

Significant differences were observed between groups in the distance walked during the 6MWT (F(2,81) = 240.19, *p* < 0.001, η^2^p = 0.86), the average step duration (F(2,81) = 16.74, *p* < 0.001, η^2^p = 0.29), and the average step length (F(2,81) = 57.92, *p* < 0.001, η^2^p = 0.59). Both the distance covered and the average step length were greater in the HPP compared to both the IPP and the LPP groups (*p* < 0.001) and in the IPP compared to the LPP groups (*p* < 0.001). The average step duration was longer in the LPP compared to both the IPP (*p* < 0.01) and HPP groups (*p* < 0.001). No significant difference was observed between the IPP and HPP groups (*p* = 0.13) (Figure 4e–g).

#### 3.3.3. Physical Performance Equation Obtained from the Functional and Biomechanical Parameters Measured

Clustering the participants into the three physical performance groups from the functional and biomechanical parameters measured generated a physical performance equation:

Physical performance = number of STS × (−0.014) + vertical acceleration of STS × 0.010 + distance covered × 0.009 + step length × (−0.098) + step duration × 0.051 + TUG time fast × 0.002 + TUG time comfort × (−0.045) −2.265

The physical performance score determined with the K-means clustering was signif-icantly different between the three groups (F(2,81) = 256.42, *p* < 0.001, η^2^p = 0.86). The score was lower in the LPP group (0.23 ± 0.35, *p* < 0.001) compared with the IPP (1.13 ± 0.18) and the HPP (2.01 ± 0.29) groups. The score in the IPP group was also lower than the HPP group (*p* < 0.001).

## 4. Discussion

The main objective of this study was to assess the functional physical capacities of older adults through the measuring of six functional and biomechanical parameters collected using smart eyeglasses embedded with an IMU (i.e., maximal vertical acceleration during the STS, number of STS, average step duration and length during the 6MWT, and TUG time in comfort and fast conditions). To the best of our knowledge, this is the first study to assess functional and biomechanical parameters with smart eyeglasses in older adults. As expected, the frailty or faller status of the participants, based on the accepted definitions used in the literature, is not sufficient to fully characterize the population tested with the measures performed. In contrast, the clustering-based approach applied in this study (i.e., K-means) allowed us to categorize the population into three groups of physical performance. With this approach, the selected functional and biomechanical parameters differed between groups independently of the participants’ frailty or faller status. However, even though the faller status did not differ among performance groups, the LPP group had a higher percentage of frail participants compared to both the IPP and the HPP group.

Based on the question, “have you fallen in the last 12 months?” [16], thirty-four of our older adults (i.e., ~40%) were considered fallers. From the data collected, only one out of the seven functional and biomechanical parameters was different between the fallers and the non-fallers (i.e., step length measured during the 6MWT). The faller group had a smaller step length than the non-faller group (Table 1). Although this result is consistent with the literature [40,41], a recent study pointed out that gait spatiotemporal parameters alone are not sufficient to identify fall risk in older adults [42]. It is well recognized that falls are multifactorial [24,43] and it is extremely challenging to determine the most accurate combination of multiple measures of risk [22]. Consequently, the functional and biomechanical parameters recorded with our smart eyeglasses do not provide a satisfactory characterization of the population tested based on the “fall” definition chosen in the present study. In addition, although the participants’ ability to remember a fall was thoroughly assessed, it cannot be excluded that the participants voluntarily or involuntarily omit the fact that they have fallen within the last 12 months and would partially bias our observations. 

Considering that frailty is associated with falls in older adults [14,15], the present study also aimed to characterize the influence of frailty status on the functional and biomechanical parameters measured. In our population, 18%, 53.5%, and 28.5% participants were considered frail, pre-frail, and robust, respectively, but lower frailty status was not associated with a higher percentage of fallers. Although no differences were observed between the frail and pre-frail groups, five of the seven functional and biomechanical parameters were better in the robust group compared to the frail group (i.e., number and average maximal vertical acceleration of STS, distance covered and step length during the 6MWT, and TUG time in fast condition (Table 2)). Similar to the results obtained for the “fall” definition, the parameters measured do not discriminate frail from pre-frail participants. However, most of the parameters were useful in differentiating frail and robust participants. Regarding the spatiotemporal parameters measured during the 6MWT, step duration did not differ between groups, although this parameter was previously reported as a relevant parameter of frailty [44,45]. In contrast, we observed a significant difference in step time [46] and 6MWT performance [47] between the robust and frail groups. The two variables measured during the 30 s STS test with the smart eyeglasses (i.e., number and average maximal vertical acceleration), that differentiate robust and frail groups, are in agreement with previous observations comparing frail older adults and middle-aged healthy participants [48]. Finally, the TUG time achieved in the fast condition permitted the distinction between frail and robust participants [49,50]. Collectively, these observations indicate that the parameters measured could be used to distinguish frail and robust participants, but were not sensitive enough to distinguish consecutive frailty phenotypes (i.e., robust from pre-frail and pre-frail from frail).

Similar to a previous study using a clustering-based approach to identifying clinical patterns associated with frailty in a population [26], the unsupervised analysis based on the parameters measured categorized our older adults into three distinct groups. Since we performed measures assessing functional physical capacities, the three groups created represent three physical performance groups (i.e., LPP, IPP, and HPP). Although most of the measured parameters differed between the three physical performance groups, some exceptions were observed between two consecutive groups (i.e., LPP with IPP or IPP with HPP), emphasizing the non-linearity and multifactorial clinical evolution of aging. One of the most important results of the present study is that all functional and biomechanical parameters measured differed between the LPP and HPP groups, with the HPP displaying a better performance than the LPP. In addition, it is worth mentioning that the proportion of fallers did not differ between these two groups. This result is consistent with a recent study showing that older adults with a higher functional capacity performed better than their counterparts with a lower functional capacity, independently of their faller status [42]. Similarly, here, we do not observe a significant difference in the proportion of fallers between the LPP and the HPP groups (Table 3). In contrast, the proportion of frail participants was lower in the HPP (8.7%) compared to the LPP group (40.9%), which is in agreement with a recent study using a hierarchical cluster analysis to classify participants into groups of similar characteristics regarding functional performance [51]. In addition, the age was different between the HPP group and the LPP group [42], the participants of the HPP group being younger. Unlike other studies using a clustering-based approach [26,27], we did not include the age of the participant, a possible predictor of our observations, as a variable in the K-means algorithm. Actually, although taken into account in these two studies, significant differences in age were still reported between the highest and lowest frail [26] or fall risk [27] groups as determined via clustering analysis. Altogether, these observations emphasized the fact that age has a negative impact on the decline in physical capacities, whether or not it is considered as a variable in a clustering-based approach. However, other studies did not report a significant influence of age on the frailty status of their participants [51,52]. Rather, based on the World Health Organization approach to healthy aging focusing on “function” [53], Dapp et al. [52] recently highlighted that a hierarchical arrangement of functional physical levels is more determinative than sex or age in describing community-dwelling persons aged 70 years and over. Along with this study, our results provide evidence that the functional and biomechanical parameters investigated could be used in older adults to discriminate low versus high levels of functional physical performance based on movements encountered in daily life activities and that six of these parameters can be collected using smart eyeglasses embedded with an IMU.

The outcomes observed for two adjacent groups (i.e., LPP with IPP and IPP with HPP) slightly differed from those reported between the LPP and HPP groups. First, and importantly, the proportion of fallers based on fall history was not significantly different between the three groups. Second, the LPP group had a greater proportion of frail participants (40.9%) compared to the IPP (10.3%), but no difference was noted between the IPP and HPP groups. Third, the age of the participants was similar for the LPP and IPP groups, whereas the participants of the IPP group were older than those of the HPP group (Table 3). From the functional and biomechanical parameters measured, six were significantly better in the IPP group compared to the LPP group (i.e., only the average maximal vertical acceleration of the STS did not differ between groups). The comparison of the IPP and HPP groups underlined a significant difference in five parameters (i.e., the number of STS performed and step duration during the 6MWT were similar in these groups). These observations have important clinical implications. For example, during the 30 s STS test, a low number of Sit-to-Stand movements achieved (i.e., <9) clearly indicates a low level of physical performance, whereas a high value of average maximal vertical acceleration (i.e., >14 m·s^−2^) indicates a high level of physical performance. In addition, the step length and the distance covered during the 6MWT, as well as the time to perform a TUG test, are the common variables that could discriminate the three levels of physical performance. Although important to screen, fall history was not related to the categorization of the population in the different physical performance groups. However, being identified as a frail individual using Fried’s frailty phenotype could indicate a higher probability of having a low level of physical performance, but we suggest complementing this evaluation with physical functional testing. Overall, the results of the present study expand our comprehensive understanding of functional decline with aging. 

A strength of this study was its categorizing of a population of older adults through a clustering-based approach using functional and biomechanical variables measured during clinical tests. Although this approach has been used recently [26,27], to the best of our knowledge, this is the first time that physical performance groups were created on physical parameters measured with an IMU integrated into smart eyeglasses. We also obtained a physical performance equation, based on the selected functional and biomechanical parameters, that is able to discriminate three different groups of physical performance among older adults. The clinical meaningfulness is that the physical performance level was determined from clinical tests and this study conforms to “Healthy Aging”, which is defined by the World Health Organization “as the process of developing and maintaining the functional ability that enables well-being in older age”. Finally, this approach incorporating categorizations by physical performance levels is important in assessing the influence of physical activity programs in older adults.

This study has some limitations. First, and although many recommendations have been addressed, this study did not strictly include all the items listed in the STROBE statement for observational studies. Second, only older adults able to walk without aids and independent in their daily movements were included, which limits the generalization of our observations. Thus, the categorization and the equation obtained are specific to our population, which included 70% female participants. Third, uncontrolled potential confounders, such as nutritional status, previous injury history, psychological factors (e.g., namely “fear of falling”, especially for those who had already fallen), and differences in physical activity levels, may have contributed to the performances in each clinical test. One further limitation lies in the completion of the clinical tests performed during a single test session with standardized instructions (e.g., arms crossed over the chest during the 30 s STS test) in a laboratory environment. The data obtained are dependent on the participant’s physical fitness at the time of testing. However, this form of testing reflects the current practice in clinical settings. Fifth, only the K-means clustering algorithm was considered in this study for categorizing the assessed population. Thus, future research needs to examine different clustering-based approaches, as well as a larger cohort of older adults. Finally, since only smart eyeglasses were used, we cannot ascertain this sensor’s superiority compared to other sensors located over the body. Nevertheless, considering that smart eyeglasses are a unobtrusive everyday object worn by more than 90% of people aged 60 years and over [54], and are well-accepted by older adults [37], their implementation in this population seems relevant and very promising.

This study is intended to be innovative and has several future prospects. First, it would be interesting to assess a larger cohort of participants with a wider age range to confirm the relevance of the functional and biomechanical parameters tested. Second, another very interesting possibility would be to record the functional and biomechanical parameters in ecological situations such as during daily life movements (e.g., at participants’ homes). Third, performing a longitudinal study would be interesting for monitoring the evolution of the physical performance of older adults over time while prospectively recording falls (i.e., the smartphone mobile application already includes a fall detection alert system). Indeed, longitudinal studies are essential for understanding the physical performance changes associated with aging and smart eyeglasses would be useful in such an experimental design. Fourth, through the smartphone mobile application developed, smart eyeglasses facilitate the remote monitoring of physical performance, allowing healthcare professionals and/or relatives to follow older adults’ physical performance changes. This remote monitoring is particularly beneficial for older adults living alone or those who have limited access to healthcare facilities. Finally, smart eyeglasses have been used and validated for human activity recognition purposes [55]. They can provide real-time feedback on physical performance, allowing older adults to adjust their movements and promoting positive changes in physical activity behaviors and performances.

## 5. Conclusions

Our study showed that a clustering-based approach to functional and biomechanical parameters measured with smart eyeglasses during clinical tests was more relevant in categorizing older adults in distinct physical performance groups than a classification based on their frailty or faller status. We have highlighted that four of the measured parameters (i.e., the step length and the distance covered during the 6MWT and the time to perform a TUG test in comfort and fast conditions) distinguished the three levels of physical performance and all the selected parameters distinguished the low and high physical performance groups. Being able to determine the physical performance levels with smart eyeglasses through movements encountered in daily life activities is very promising as a way of monitoring an older adult population in ecological conditions. With the implementation of machine learning techniques [56] and the use of smart eyeglasses, it may be possible to identify a potential decline in physical performance and, ideally, to prevent falls.

## Figures and Tables

**Table 1:** Functional and biomechanical parameters obtained in the faller and in the non-faller groups based on fall history.

	Faller Group n = 34	Non-Faller Group n = 50	*p* Value
STS: Number in 30 s	10.82 ± 3.42	11.10 ± 3.44	0.72
STS: Average maximal vertical acceleration (m·s^−2^)	13.02 ± 2.15	12.84 ± 2.04	0.69
6MWT: Distance covered (m)	410.36 ± 80.69	441.35 ± 70.93	0.07
6MWT: Average step duration (s)	0.48 ± 0.04	0.48 ± 0.05	0.56
6MWT: Average step length (m)	0.65 ± 0.12	0.71 ± 0.12	**0.04**
TUG: Time comfort (s)	8.87 ± 1.54	9.08 ± 1.73	0.55
TUG: Time fast (s)	7.27 ± 1.74	7.06 ± 1.77	0.54

STS: Sit-to-Stand; 6MWT: six-minute walking test; TUG: Timed Up and Go test. Mann–Whitney U test was applied for TUG Time fast and ANOVAs were used for all other parameters. *p* value in bold represents a significant difference between the faller and non-faller groups.

**Table 2:** Functional and biomechanical parameters obtained in the robust, pre-frail, and frail groups based on Fried’s frailty phenotype.

	Robust Group n = 24	Pre-Frail Group n = 45	Frail Group n = 15
STS: Number in 30 s	12.79 ± 3.12 **	10.82 ± 2.79	8.60 ± 4.12
STS: Average maximal vertical acceleration (m·s^−2^)	13.95 ± 1.27 **	12.82 ± 2.13	11.53 ± 2.17
6MWT: Distance covered (m)	452.76 ± 73.39 **	434.21 ± 64.04	374.25 ± 91.33
6MWT: Average step duration (s)	0.48 ± 0.05	0.48 ± 0.04	0.50 ± 0.04
6MWT: Average step length (m)	0.72 ± 0.10 *	0.69 ± 0.12	0.62 ± 0.15
TUG: Time comfort (s)	8.39 ± 1.36	9.14 ± 1.81	9.54 ± 1.34
TUG: Time fast (s)	6.64 ± 1.44 *	7.11 ± 1.74	8.09 ± 1.95

STS: Sit-to-Stand; 6MWT: six-minute walking test; TUG: Timed Up and Go test. Kruskall–Wallis test was applied for fast TUG and ANOVAs were performed for all other parameters. * and ** indicate significant differences between the robust and the frail groups (*p* < 0.05 and *p* < 0.01, respectively).

**Table 3:** Distribution, demographic data, fall history, and Fried’s phenotype of participants in the low physical performance (LPP), intermediate physical performance (IPP), and high physical performance (HPP) groups.

	LPP	IPP	HPP
n	22	39	23
Sex (% females)	68%	72%	65%
Age (years)	77.45 ± 4.39 ***	75.12 ± 5.61 ***	69.43 ± 4.29
Height (cm)	164.73 ± 8.15	164.54 ± 7.83	168.61 ± 8.57
Body mass (kg)	68.52 ± 14.76	69.16 ± 13.52	68.94 ± 12.97
Fallers (%)	45.45	41.03	34.78
Robust (%)	18.18	25.64	43.48
Pre-frail (%)	40.91	64.10	47.82
Frail (%)	40.91	10.26 ^££^	8.70 ^££^

ANOVAs were applied on demographic data and k proportions tests were used for sex, fall history, and Fried’s phenotype comparisons. *** indicates a significant difference with the HPP group (*p* < 0.001). ^££^ indicates a significant difference with the LPP group (*p* < 0.01).

## Data Availability

The data presented in this study are available on request from the corresponding author.
